# Association between the oxidative balance score and gynecologic cancers from the National Health and Nutrition Examination Survey: An observational study

**DOI:** 10.1097/MD.0000000000044782

**Published:** 2025-09-26

**Authors:** Ya'nan Yan, Lei Zhang, Shuai Yuan, Ting Zhang, Can Shi

**Affiliations:** a Department of Gynecology, The Affiliated Huai’an No. 1 People’s Hospital of Nanjing Medical University, Huai’an, Jiangsu, China.

**Keywords:** gynecological cancer, multivariate logistic model, National Health and Nutrition Examination Survey, oxidative balance score

## Abstract

This study aimed to examine the potential correlations between the oxidative balance score (OBS) and gynecological cancers susceptibility. A cross-sectional design was employed, utilizing datasets from the National Health and Nutrition Examination Survey spanning 2 decades from 1999 through 2018. Our investigation demonstrated that OBS exhibited significant protective correlations with gynecologic cancers (odds ratio [OR]=0.985, 95% confidence interval [CI]: 0.974–0.997, *P*=.011), specifically cervical cancer (OR=0.974, 95% CI: 0.952–0.996, *P*=.019) and ovarian cancer (OR=0.950, 95% CI: 0.912–0.990, *P*=.015). The protective correlations of OBS with gynecologic, cervical, and ovarian cancers displayed a dose–response relationship with elevated OBS levels, yielding ORs of 0.765 (95% CI: 0.614–0.952, *P*=.017), 0.474 (95% CI: 0.289–0.777, *P*=.003), and 0.397 (95% CI: 0.167–0.942, *P*=.036), respectively, in the uppermost quartile. The OBS demonstrates significant protective correlations with gynecological cancers, with notably marked effects identified in cervical and ovarian cancers. These observations provide innovative perspectives regarding the pathogenesis of gynecological neoplasms and carry important ramifications for clinical prevention and public health interventions.

## 1. Introduction

Gynecological cancers pose significant threats to the health of women worldwide. Within this spectrum, breast, cervical, ovarian, and uterine cancers constitute critical entities.^[1,2]^ Breast cancer continues to represent the predominant malignant tumor affecting women worldwide, registering roughly 2.3 million newly diagnosed cases alongside 666,000 fatalities throughout 2022. Furthermore, low- and middle-income regions have witnessed substantially accelerated growth rates in breast cancer incidence compared to high-income countries during recent decades.^[3]^ Cervical cancer constitutes a malignancy predominantly attributable to chronic infection with oncogenic human papillomavirus variants. This condition ranks as the most commonly identified cancer across 25 nations and represents the primary source of oncological mortality among female populations in 37 countries.^[1,3]^ Ovarian malignancy exhibits unfavorable prognostic outcomes and persists as a significant contributor to female oncological mortality internationally, distinguished by its insidious early-stage manifestations and propensity for delayed detection.^[3,4]^ Uterine cancer likewise poses substantial health challenges, documenting 417,000 new diagnoses and 97,000 fatalities during 2022, emphasizing its considerable life-threatening consequences.^[3]^ Moreover, the global disease burden of these 4 cancers have increased at heterogeneous rates from 1990 to 2021.^[5]^ Therefore, strengthening the prevention, early diagnosis and treatment of gynecological cancers is highly important for reducing the disease burden and improving women’s health.^[6]^

An important link in the pathogenesis of cancer is an imbalance of oxidants and antioxidants, which is also called oxidative stress.^[7,8]^ It usually leads to the onset and development of cancer by inducing damage to proteins, DNA, lipids, and organelles.^[9,10]^ The organism necessitates redox equilibrium for sustaining critical physiological processes, with dysregulation now recognized as a fundamental contributor to numerous prevalent pathologies, including metabolic disorders, Alzheimer disease, intervertebral disc deterioration, and degenerative joint disease.^[11–14]^ Specific dietary constituents, particularly lipids and iron, function as pro-oxidative agents promoting oxidative stress. Conversely, various nutrients serve as antioxidative elements that diminish oxidative system activity, encompassing cobalamin, ascorbic acid, tocopherol, and calcium. Likewise, certain behavioral patterns, including tobacco consumption and alcohol intake, exhibit pro-oxidative properties.^[15]^ Within practical contexts, the simultaneous presence of oxidative and antioxidative factors generates intricate biological interactions, complicating investigations into health implications of individual pro-oxidant/antioxidant agents.^[16]^ Consequently, investigators have established an innovative systemic oxidative stress metric, the oxidative balance score (OBS), to evaluate comprehensive oxidative stress conditions through consideration of both nutritional and behavioral pro-oxidant/antioxidant factors.^[15,17]^ Multiple investigations have examined OBS effects on health outcomes, including cardiovascular pathology, nonalcoholic hepatic steatosis, and periodontal health.^[18–21]^ And OBS has been proven to be associated with a significantly reduced risk of all-cause mortality and cancer-specific mortality among cancer survivors.^[22]^

Additionally, numerous investigations have explored correlations between OBS and malignancies. For instance, both case-control and cohort investigations have demonstrated significant inverse relationships between elevated OBS and colorectal cancer.^[23,24]^ Two case-control investigations revealed no association between OBS and prostatic cancer risk.^[25,26]^ In terms of female specific cancers, research has focused only on breast cancer, and all these studies were based on the construction of OBSs with parts of oxidants and antioxidants.^[27,28]^ This consequently resulted in inadequate holistic evaluations of oxidative stress while impeding thorough elucidation of oxidative dysregulation’s involvement in gynecological malignancy pathogenesis and progression. Furthermore, there is currently a lack of research exploring the relationship between OBS and gynaecological tumors. Therefore, to address this significant problem, it is necessary to conduct a scientific study to assess the relationship between OBS levels and gynaecological tumors in the female population. This research would also provide scientific evidence for the use of lifestyle interventions in the prevention and prognosis of gynaecological tumors, as well as offer important insights into the general mechanisms by which OBS influences cancer.

Accordingly, our investigation employed contemporary OBS computational methodologies to systematically examine associations between OBS and gynecological cancers (encompassing breast, cervical, ovarian, and uterine cancers) utilizing the National Health and Nutrition Examination Survey (NHANES) database. This research endeavored to illuminate the distinctive contributions of oxidative stress throughout gynecological cancers initiation and advancement, thereby establishing foundational evidence for prophylactic strategies and early intervention approaches targeting gynecological malignancies.

## 2. Materials and methods

### 2.1. Study design and population

This investigation utilized data from NHANES, a continuous, nationwide health surveillance program administered by the National Center for Health Statistics. The survey gathered extensive information encompassing physical examinations, laboratory assessments, nutritional interviews, and sociodemographic characteristics across all age groups within the U.S. population, intending to characterize the nutritional and health profile of Americans. The program functioned through biennial cycles, providing publicly available datasets for scientific inquiry. Our analysis incorporated information from 10 consecutive cycles (spanning 1999–2018) involving adult female subjects to investigate associations between OBS and gynecologic cancers, encompassing breast, cervical, ovarian, and uterine cancers.

**Figure 1.**
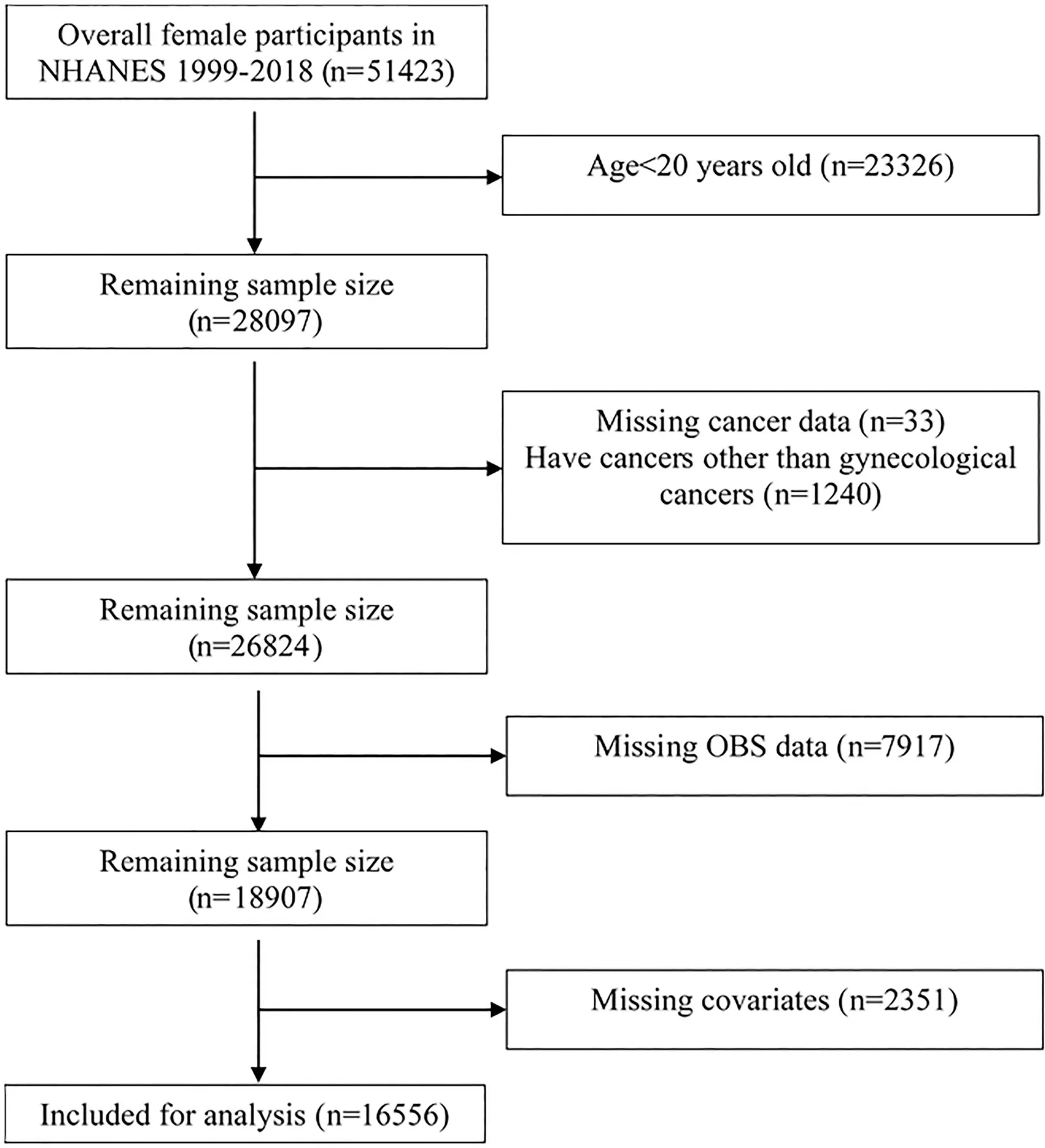
Flowchart of the sample selection from NHANES 1999–2018. NHANES = National Health and Nutrition Examination Survey.

The inclusion and exclusion criteria for specific data are shown in Figure 1. In summary, a total of 16,556 samples were included for analysis, among which 454, 215, 72, and 133 cases were diagnosed with breast, cervical, ovarian, and uterine cancers, respectively, and 874 cases were diagnosed with gynecological cancers in total.

### 2.2. Exposure definition

OBS represents a holistic metric evaluating an individual’s comprehensive oxidative/antioxidative equilibrium.^[20]^ This metric encompasses 20 constituent elements, incorporating 16 nutritional factors and 4 behavioral factors. The nutritional factors comprise 14 antioxidative constituents (encompassing dietary fiber, carotenoids, riboflavin, niacin, pyridoxine, total folate, cobalamin, ascorbic acid, tocopherol, calcium, magnesium, zinc, copper, and selenium) alongside 2 pro-oxidative constituents (total lipids and iron), whereas the behavioral factors incorporate 1 antioxidative element (physical exercise) and 3 pro-oxidative elements (ethanol consumption, body mass index (BMI), and cotinine concentrations). Each OBS element received scoring allocations of 0, 1, or 2 following established protocols. Regarding antioxidative constituents, subjects underwent stratification into tertiles representing low, intermediate, and high categories, receiving scores of 0, 1, and 2 correspondingly. Conversely, pro-oxidative constituents employed inverse scoring methodology. Subjects underwent tertile-based stratification into low, intermediate, and high categories, receiving scores of 2, 1, and 0 correspondingly. Notably, ethanol consumption followed distinct scoring parameters, utilizing thresholds of 15 g/day and 0 g/day to classify subjects as heavy drinkers, non-heavy drinkers, and nondrinkers, with respective scores of 0, 1, and 2. Physical activity underwent categorization into low, intermediate, and high groups employing thresholds of 400 and 1000 MET-min/week, with corresponding scores of 0, 1, and 2. Individual OBS values represented the cumulative total across all 20 constituent scores.

### 2.3. Covariates

Based on previous studies,^[29,30]^ this study included the following 10 covariates: age, race, family poverty ratio, education level, marital status, smoking status, alcohol status, BMI, hypertension, and diabetes mellitus.

### 2.4. Outcome definition

Utilizing the questionnaire item “Ever told you had cancer or malignancy?” we established cancer presence. Subsequently, through the questionnaire item “What kind of cancer?” we ascertained diagnosis of breast, cervical, ovarian, or uterine malignancies.^[31]^ Subjects diagnosed with any aforementioned malignancy were classified as having gynecologic cancers.

### 2.5. Statistical analysis

#### 2.5.1. Descriptive statistics

The continuous covariates included in this analysis did not conform to normal distributions. Therefore, they were presented using medians and interquartile ranges. Subsequently, the Wilcoxon rank-sum test was employed for intergroup difference evaluations. Categorical parameters were expressed as frequencies and percentages, with χ^2^ test utilized for intergroup comparisons.

#### 2.5.2. Association analysis

Initially, univariate logistic regression methodology was implemented to investigate correlations between OBS and cumulative gynecologic malignancy risk alongside their individual subtypes. This preliminary analysis, designated Model 1, quantified effect magnitudes through odds ratios (ORs) with corresponding 95% confidence intervals. Following this, 3 distinct multivariate logistic regression frameworks were developed incorporating progressive covariate adjustments. The specifications for these analytical models were delineated as follows. Model 2 incorporated adjustments for age, race, family poverty ratio, education level, and marital status. Model 3 expanded upon Model 2 through additional adjustments for smoking status, alcohol status and BMI. Model 4 built upon Model 3 with subsequent adjustments for hypertension and diabetes mellitus status. Additionally, OBS was converted into an ordered categorical variable with 4 categories using quartiles as cutoff values, and it was included in the aforementioned models to examine the relationships between different levels of OBS and the total risk of gynecologic malignancies and their subtypes. Furthermore, the trends of these effects were analyzed.

We also performed stratified analyses to investigate the effects of OBS in different populations. Throughout this procedure, age underwent dichotomization utilizing a 50-year threshold, while BMI was stratified into higher and lower categories based on median values. Subsequently, interaction analyses were performed investigating potential interplay between OBS and designated covariates. Statistical computations were executed utilizing R software, establishing *P* < .05 as the significance criterion.

## 3. Results

Table 1 presented the baseline characteristics differentiating subjects with and without gynecological malignancies. Statistically significant OBS variations emerged between cohorts (*P* < .05). Our analysis revealed that, relative to the non-gynecological malignancy cohort, the gynecological malignancy cohort demonstrated comparatively elevated proportions of elderly subjects, Non-Hispanic White individuals, and those categorized as living independently (*P* < .05). Furthermore, the gynecological malignancy cohort exhibited comparatively increased proportions of subjects with smoking histories, hypertensive conditions, and diabetic status, while demonstrating reduced alcohol consumption prevalence (*P* < .05). No statistically meaningful variations manifested regarding household poverty index, educational attainment, and BMI (*P* > .05). Tables S1–S4 (Supplemental Digital Content, https://links.lww.com/MD/Q141) delineated baseline characteristics across the 4 gynecological malignancy subtypes. Statistically significant OBS differences emerged within cervical and ovarian cancer groups (*P* < .05), whereas breast and uterine cancer groups exhibited no significant differences (*P* > .05).

**Table 1:** Baseline characteristics of participants with and without gynecological cancers.

Variables	Non-Gynecological cancers (N = 15,682)	Gynecological cancers (N = 874)	*P* value
OBS	17.00 (12.00, 23.00)	16.00 (12.00, 22.00)	.004
Age (yr)	46.00 (32.00, 61.00)	62.00 (49.00, 73.00)	<.001
Race/ethnicity, N			
Mexican American	2809 (17.91%)	99 (11.33%)	<.001
Non-Hispanic Black	3298 (21.03%)	119 (13.62%)	
Non-Hispanic White	6950 (44.32%)	545 (62.36%)	
Other Hispanic	1331 (8.49%)	61 (6.98%)	
Other Race	1294 (8.25%)	50 (5.72%)	
Family poverty ratio, N			
Low	4841 (30.87%)	257 (29.41%)	.639
Median	5906 (37.66%)	333 (38.10%)	
High	4935 (31.47%)	284 (32.49%)	
Education level, N			
Below high school	3655 (23.31%)	206 (23.57%)	.425
High school	3446 (21.97%)	207 (23.68%)	
Above high school	8581 (54.72%)	461 (52.75%)	
Marital status, N			
Married	8320 (53.05%)	436 (49.89%)	.001
Living with partner	1123 (7.16%)	43 (4.92%)	
Living alone	6239 (39.78%)	395 (45.19%)	
Smoking status, N			
Yes	5519 (35.19%)	420 (48.05%)	<.001
No	10,163 (64.81%)	454 (51.95%)	
Alcohol status, N			
Yes	8601 (54.85%)	447 (51.14%)	.033
No	7081 (45.15%)	427 (48.86%)	
BMI (kg/m^2^)	28.10 (24.01, 33.30)	28.33 (24.20, 33.30)	.511
Hypertension, N			
Yes	5057 (32.25%)	450 (51.49%)	<.001
No	10,625 (67.75%)	424 (48.51%)	
Diabetes mellitus, N			
Yes	1635 (10.43%)	147 (16.82%)	<.001
No	14,047 (89.57%)	727 (83.18%)	

BMI = body mass index, OBS = oxidative balance score.

**Figure 2.**
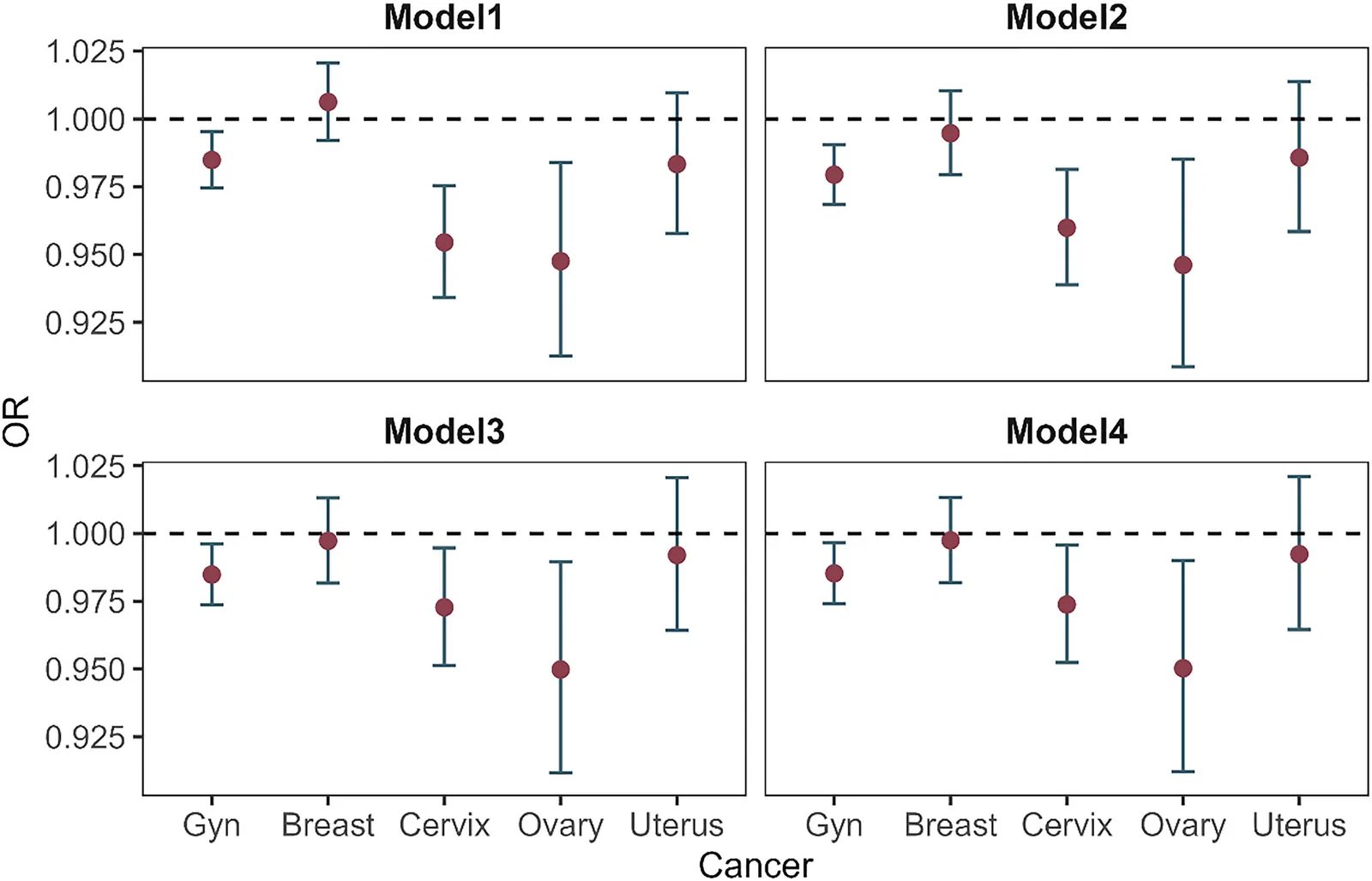
Correlations between OBS and gynecologic malignancy risk across various logistic regression frameworks. Model 1: No covariate adjustments implemented. Model 2: Adjustments incorporated for age, ethnicity, household poverty index, educational attainment, and matrimonial status. Model 3: Adjustments encompassed age, ethnicity, household poverty index, educational attainment, matrimonial status, tobacco use history, alcohol consumption, and BMI. Model 4: Comprehensive adjustments included age, ethnicity, household poverty index, educational attainment, matrimonial status, tobacco use history, alcohol consumption, BMI, hypertensive condition, and diabetic status. BMI = body mass index, Gyn = gynecologic malignancies, OBS = oxidative balance score, OR = odds ratio.

The associations between OBS and the risk of gynecologic cancers as well as their subtypes across different logistic models are illustrated in Figure 2. Univariate logistic regression revealed that OBS had protective effects on gynecologic cancers as well as cervical and ovarian cancers, with corresponding ORs of 0.985 (95% CI: 0.975–0.995, *P* = .004), 0.954 (95% CI: 0.934–0.975, *P* < .001), and 0.948 (95% CI: 0.913–0.984, *P* = .005), respectively. However, the effects of OBS on breast cancer and uterine cancer were not statistically significant. The results of the multivariate logistic regression model were similar, and the number of included covariates had little influence on the effect size of OBS. In Model 4, which included all considered covariates, OBS demonstrated protective effects on gynecologic cancers (OR = 0.985, 95% CI: 0.974–0.997, *P* = .011), cervical cancer (OR = 0.974, 95% CI: 0.952–0.996, *P* = .019), and ovarian cancer (OR = 0.950, 95% CI: 0.912–0.990, *P* = .015). Similarly, no statistically significant protective effects were observed for breast cancer (OR = 0.997, 95% CI: 0.982–1.013, *P* > .05) or uterine cancer (OR = 0.992, 95% CI: 0.965–1.021, *P* > .05).

**Figure 3.**
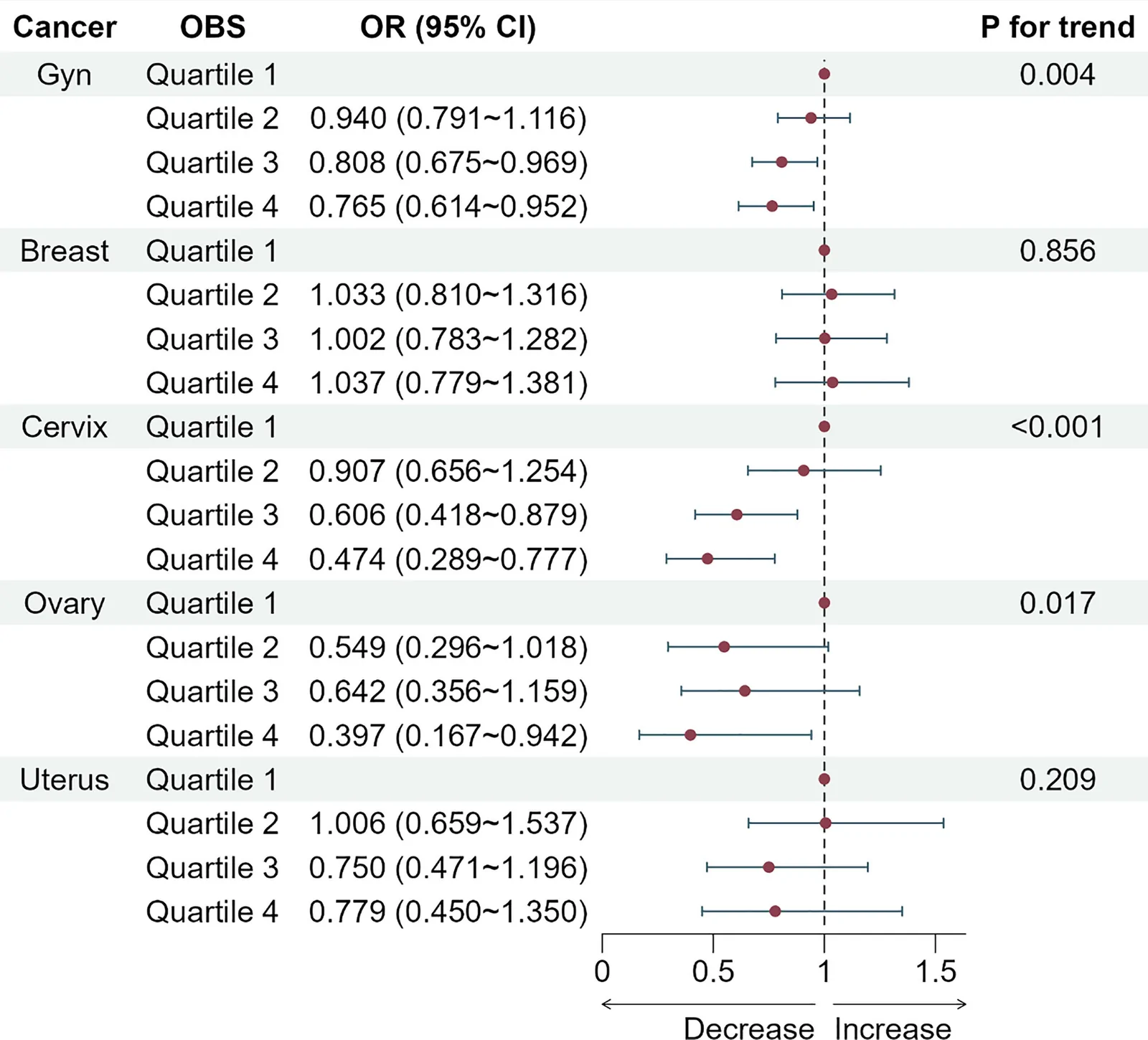
Associations between OBS quartiles and gynecologic malignancy risk. The findings derived from univariate logistic regression modeling (Model 1). OBS quartile 1 served as the reference category. BMI = body mass index, CI = confidence interval, Gyn = gynecologic malignancies, OBS = oxidative balance score, OR = odds ratio.

We subsequently evaluated correlations between OBS quartiles and gynecologic malignancy risk alongside their subtypes, with findings depicted in Figure 3. Comprehensively, the protective associations of OBS against gynecologic cancers strengthened with ascending OBS levels (*P* = .004), achieving an effect magnitude of 0.765 (95% CI: 0.614–0.952, *P* = .017) within the highest quartile. Comparable patterns emerged for cervical and ovarian neoplasms, with ORs in the uppermost quartile achieving 0.474 (95% CI: 0.289–0.777, *P* = .003) and 0.397 (95% CI: 0.167–0.942, *P* = .036) respectively. No meaningful trends materialized regarding associations between OBS quartiles and breast or uterine cancer risk (95% CI: 0.779–1.381, *P* > .05; 95% CI: 0.450–1.350, *P* > .05).

**Figure 4.**
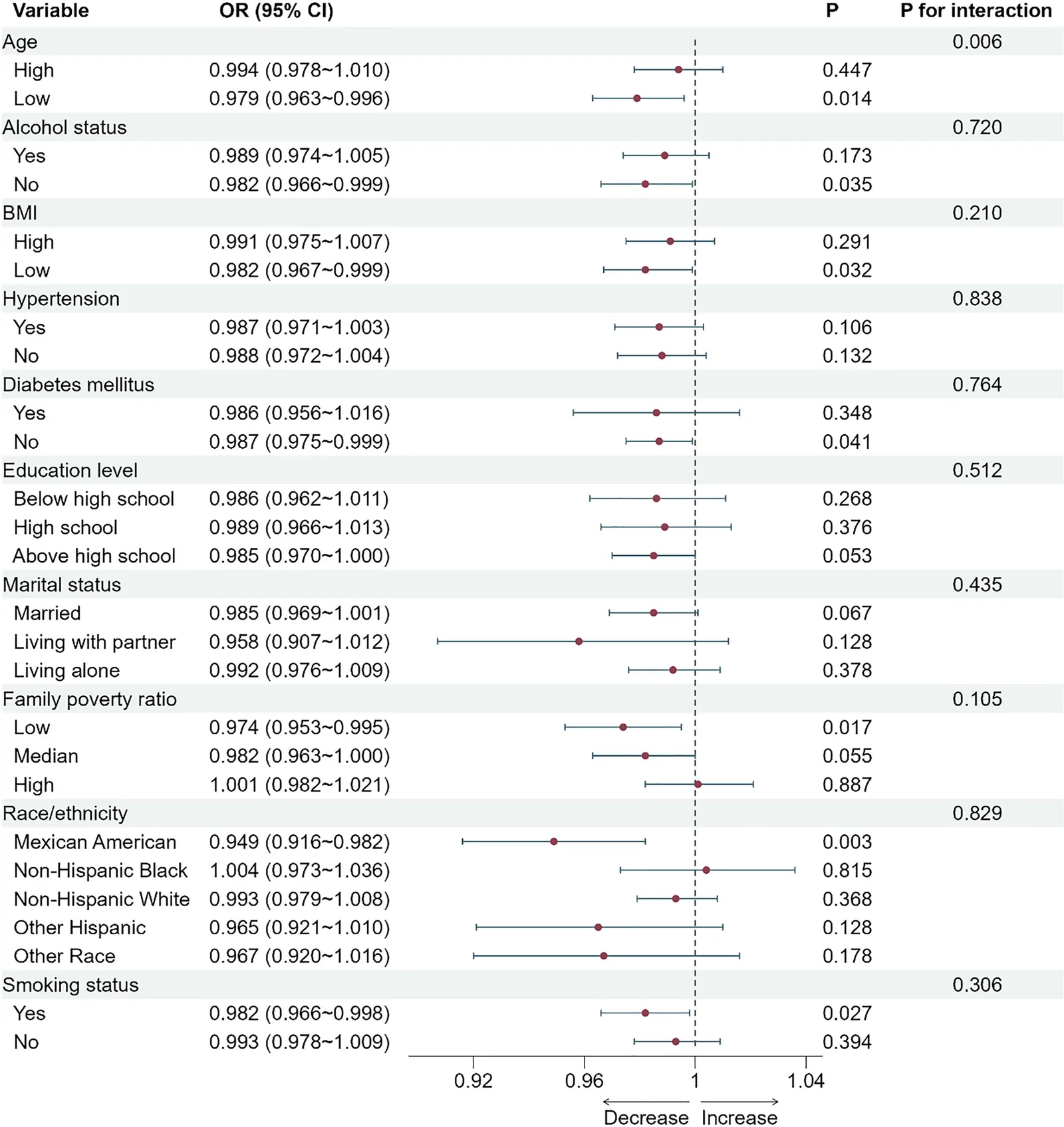
Stratified and interaction evaluations examining OBS relationships with gynecologic malignancies. Results obtained from Model 4: Incorporating adjustments for age, ethnicity, household poverty index, educational attainment, matrimonial status, tobacco use history, alcohol consumption, BMI, hypertensive condition, and diabetic status. BMI = body mass index, CI = confidence interval, Gyn = gynecologic malignancies, OBS = oxidative balance score, OR = odds ratio.

Subgroup analyses revealed enhanced protective associations of OBS against cumulative gynecologic cancers among specific populations: younger subjects, tobacco users, alcohol abstainers, individuals with lower BMI, non-diabetic participants, subjects with reduced household poverty indices, and Mexican American individuals (*P* < .05). Interaction analyses identified significant interplay exclusively between OBS and age (*P* < .05). Detailed results appear in Figure 4.

## 4. Discussion

This investigation, utilizing NHANES datasets spanning 1999 through 2018, employed contemporary OBS methodology to examine correlations between oxidative stress and gynecological malignancies, encompassing breast, cervical, ovarian, and uterine malignancies. Our findings demonstrated protective associations of OBS against gynecological cancers, predominantly manifested in cervical and ovarian cancers, with protective effects intensifying proportionally with elevated OBS levels.

Our study outcomes aligned with established literature findings. For instance, a case-control investigation in Iran demonstrated that OBS and dietary phytochemical indices exhibited capacity to diminish colorectal cancer risk among the Iranian population.^[23]^ A prospective study of older women in Iowa reported that OBS in terms of diet and lifestyle was inversely associated with colorectal cancer risk.^[24]^ Similarly, our study found that OBS has protective effects against gynecological cancers, especially in cervical and ovarian cancers. These findings suggested that in different types of cancer, the oxidative balance may influence the development of tumors through similar mechanisms.

The protective effect of OBS on gynecological cancers, especially cervical and ovarian cancers, may involve a series of complex biological mechanisms. Oxidative stress represented a fundamental mechanism in carcinogenesis and neoplastic progression. Within physiological parameters, the organism’s oxidative and antioxidative systems maintained equilibrium to preserve intracellular homeostasis.^[7]^ Disruption of this equilibrium generated oxidative stress conditions, resulting in excessive reactive oxygen species (ROS) production. ROS possessed potent oxidative capacity, enabling damage to cellular biomacromolecules including proteins, DNA, and lipids, consequently inducing multiple cellular dysfunction pathways and facilitating oncogenesis and tumor advancement.^[8]^ Regarding genotoxic effects, ROS mediated DNA strand disruption, base alterations, and mutagenic events.^[10]^ Persistent DNA damage without adequate repair mechanisms precipitated genomic instability and cellular malignant transformation, escalating carcinogenic susceptibility.^[12]^ The antioxidant components of OBS could effectively remove ROS and reduce DNA damage. Antioxidant vitamins, such as vitamin C and vitamin E, could provide electrons directly, thereby neutralizing ROS and blocking its oxidative attack on DNA.^[13]^ Minerals, such as selenium and zinc, were important components of antioxidant enzymes in the body, which participate in the antioxidant defense system and enhance the body’s ability to clear ROS.^[14]^ In addition, oxidative stress could affect cell signaling pathways.^[9]^ ROS could modify key proteins in the signaling pathways through oxidation to activate or deactivate them, thus affecting cell proliferation, apoptosis and migration.^[10]^ The antioxidant effects of OBS could inhibit these abnormally activated signaling pathways, allowing cells to resume normal physiological functions.^[12]^

Furthermore, oxidative stress exerted considerable influence on immunological function, which represented critical factors in carcinogenesis and tumor progression.^[13]^ Balanced redox conditions constituted fundamental requirements for preserving optimal immune system performance. Under conditions of elevated oxidative stress, immune cell functionality became compromised and immunosurveillance mechanisms deteriorated, facilitating tumor cell evasion from immunological recognition and elimination.^[14]^ The antioxidative constituents within OBS contributed to maintaining physiological immune function while augmenting immune cell capacity for tumor identification and cytotoxicity.^[20]^ For instance, physical activity, serving as a behavioral antioxidative element of OBS, not only enhanced systemic antioxidant capabilities but also stimulated immune cell circulation and activation, reinforced immunosurveillance mechanisms, and facilitated prompt elimination of neoplastic cells.^[21]^

Oxidative stress plays a significant role in the development and progression of gynaecological cancers. The occurrence of cervical cancer is closely associated with persistent infection by high-risk human papilloma virus (HPV).^[32]^ HPV infection influences cellular oxidative stress, leading to a series of changes such as cell proliferation and cell death, thereby promoting HPV-induced carcinogenesis. Research indicates that dietary antioxidants have a certain role in inhibiting HPV persistent infection and the progression of precancerous lesions associated with cervical cancer.^[33]^ In studies on ovarian cancer, oxidative stress has been found to cause oxidative damage, promote the development of ovarian cancer, and even lead to drug resistance.^[34,35]^ Antioxidant levels are associated with the severity of ovarian cancer, and OBS just reflects a comprehensive score of antioxidant intake and lifestyle factors, which aligns with the observation in this study that higher oxidative stress scores are significantly associated with reduced risks of ovarian and cervical cancer.

It is worth noting that in this study, OBS did not show a significant protective effect in breast cancer and endometrial cancer. This maybe because the pathogenesis of breast cancer and endometrial cancer is influenced by multiple factors such as hormone levels, insulin resistance, and metabolic syndrome, while the antioxidant levels reflected by OBS may not be the dominant factor. Research indicates that breast cancer is a heterogeneous disease, with primary risk factors involving genetic susceptibility, hormonal factors, reproductive history, and lifestyle.^[36–38]^ Endometrial cancer is also a hormone-induced disease.^[39,40]^ Therefore, even if high OBS can mitigate some oxidative damage, its protective effects may be overshadowed by other factors, leading to non-significant associations in this study. Additionally, the OBS score itself has certain limitations, such as its inadequate reflection of individual antioxidant enzyme systems and genetic susceptibility.

Based on large-scale and national NHANES database, this study could reflect the overall situations of adult women in the United States and provided good representation, thus the results of this study had certain universality and promotion value. Meanwhile, this study adopted the latest OBS calculation method, which considered a variety of oxidative/antioxidant factors in diet and lifestyle and assessed the oxidative stress state of the body comprehensively, making our study more scientific and accurate. Moreover, this study included a variety of covariates for adjustment, reducing the impact of confounding factors on the results and improving the reliability of the results.

The results of this study had important clinical and public health significance. In clinical practice, this information could help doctors gain deeper understandings of the pathogenesis of gynecological tumors, providing opportunities to develop personalized prevention and treatment programs for patients. For example, women with low OBS may be advised to adjust their diet and lifestyle to increase their intake of antioxidant foods while strengthening physical activity to increase OBS levels and reduce cancer risk. In the course of cancer treatment, monitoring the OBS level of patients is helpful for evaluating the treatment effect and prognosis. From public health perspectives, the results of this study provide a scientific basis for the development of cancer prevention for women. Health education can improve women’s understanding of the relationship between oxidation balance and cancer, promote the popularization of healthy lifestyles, and effectively reduce the incidence of gynecological cancers.

Nevertheless, several limitations warrant consideration in our investigation. In this study, no significant statistical association was found between BMI and breast cancer. Although many studies support a strong association between high BMI and the incidence of breast cancer, multiple multicentre studies have shown inconsistent results regarding the risk of breast cancer associated with BMI before and after menopause.^[41,42]^ Additionally, some studies suggest that metabolic markers and body composition may provide a more comprehensive prediction of breast cancer risk than BMI alone.^[43]^ Furthermore, this study is a retrospective study based on the NHANES database and may be subject to bias. Although the study adjusted for some covariates, it did not include details such as physical activity levels or dietary patterns, which may interfere with the effects of BMI through inflammatory or metabolic pathways. This underscores the need for further research to validate these findings.

Given the cross-sectional design, determining causality between OBS and gynecological malignancies proved challenging, permitting only associative observations. Furthermore, although numerous covariates were incorporated, residual confounding variables, including unexplored environmental determinants and genetic predispositions, could potentially influence result accuracy. Additionally, the weighting allocation for individual components within the OBS computational framework may involve subjective determinations, with alternative weighting schemes potentially yielding differential outcomes.

In summary, through the analysis of NHANES data, this study revealed that OBS has protective effects on gynecological cancers, especially in cervical and ovarian cancers. These findings offer novel insights into the etiopathogenesis of gynecological malignancies and carry substantial implications for clinical prophylaxis and public health interventions. Subsequent investigations should implement prospective cohort methodologies to establish definitive causal relationships between OBS and gynecological cancers, thereby elucidating the contribution of oxidative equilibrium in gynecological cancers with enhanced precision.

## Acknowledgments

The authors sincerely thank the NHANES and related workers for the collection and management of large-scale data resources.

## Author contributions

**Data curation:** Ya'nan Yan, Lei Zhang, Shuai Yuan, Ting Zhang.

**Formal analysis:** Ya'nan Yan, Lei Zhang, Shuai Yuan.

**Funding acquisition:** Can Shi.

**Methodology:** Ya'nan Yan, Lei Zhang, Shuai Yuan, Ting Zhang, Can Shi.

**Resources:** Can Shi.

**Software:** Ya'nan Yan, Lei Zhang, Shuai Yuan.

**Writing – original draft:** Ya'nan Yan.

**Writing – review & editing:** Can Shi.

## Supplementary Material
