# Supplementary material for: Association between the oxidative balance score and gynecologic cancers from the National Health and Nutrition Examination Survey: An observational study
Source: Medicine (Baltimore). 2025 Sep 26;104(39):e44782. doi: 10.1097/MD.0000000000044782 (PMC13593086; doi:10.1097/MD.0000000000044782)
Supplement: Supplementary file 1 [file medi-104-e44782-s001.pdf]

Supplemental Materials

Table S1 Baseline characteristics of participants with and without breast cancer.

Variables	Non-breast cancer (N = 16,102)	breast cancer (N = 454)	*P*-value
**OBS**	17.00 (12.00, 23.00)	17.00 (13.00, 23.00)	0.413
**Age (years)**	46.00 (33.00, 61.00)	69.00 (60.00, 77.00)	<0.001
**Race/ethnicity, N**			<0.001
Mexican American	2,870 (17.82%)	38 (8.37%)	
Non-Hispanic Black	3,350 (20.80%)	67 (14.76%)	
Non-Hispanic White	7,199 (44.71%)	296 (65.20%)	
Other Hispanic	1,367 (8.49%)	25 (5.51%)	
Other Race	1,316 (8.17%)	28 (6.17%)	
**Family poverty ratio, N**			<0.001
Low	5,003 (31.07%)	95 (20.93%)	
Median	6,058 (37.62%)	181 (39.87%)	
High	5,041 (31.31%)	178 (39.21%)	
**Education level, N**			0.044
Below high school	3,777 (23.46%)	84 (18.50%)	
High school	3,550 (22.05%)	103 (22.69%)	
Above high school	8,775 (54.50%)	267 (58.81%)	
**Marital status, N**			<0.001
Married	8,528 (52.96%)	228 (50.22%)	
Living with partner	1,155 (7.17%)	11 (2.42%)	
Living alone	6,419 (39.86%)	215 (47.36%)	
**Smoking status, N**			0.029
Yes	5,754 (35.73%)	185 (40.75%)	
No	10,348 (64.27%)	269 (59.25%)	
**Alcohol status, N**			0.069
Yes	8,819 (54.77%)	229 (50.44%)	
No	7,283 (45.23%)	225 (49.56%)	
**BMI (kg/m^2)**	28.13 (24.04, 33.34)	27.60 (24.10, 32.50)	0.199
**Hypertension, N**			<0.001
Yes	5,254 (32.63%)	253 (55.73%)	
No	10,848 (67.37%)	201 (44.27%)	
**diabetes mellitus, N**			<0.001
Yes	1,698 (10.55%)	84 (18.50%)	
No	14,404 (89.45%)	370 (81.50%)	

Supplemental Materials

Table S2 Baseline characteristics of participants with and without cervix cancer.

Variables	Non-cervical cancer (N = 16,341)	Cervical cancer (N = 215)	*P*-value
**OBS**	17.00 (12.00, 23.00)	15.00 (10.00, 21.00)	<0.001
**Age (years)**	46.00 (33.00, 62.00)	45.00 (35.00, 57.00)	0.376
**Race/ethnicity, N**			<0.001
Mexican American	2,884 (17.65%)	24 (11.16%)	
Non-Hispanic Black	3,394 (20.77%)	23 (10.70%)	
Non-Hispanic White	7,351 (44.99%)	144 (66.98%)	
Other Hispanic	1,379 (8.44%)	13 (6.05%)	
Other Race	1,333 (8.16%)	11 (5.12%)	
**Family poverty ratio, N**			0.002
Low	5,008 (30.65%)	90 (41.86%)	
Median	6,168 (37.75%)	71 (33.02%)	
High	5,165 (31.61%)	54 (25.12%)	
**Education level, N**			0.266
Below high school	3,807 (23.30%)	54 (25.12%)	
High school	3,598 (22.02%)	55 (25.58%)	
Above high school	8,936 (54.68%)	106 (49.30%)	
**Marital status, N**			0.004
Married	8,666 (53.03%)	90 (41.86%)	
Living with partner	1,145 (7.01%)	21 (9.77%)	
Living alone	6,530 (39.96%)	104 (48.37%)	
**Smoking status, N**			<0.001
Yes	5,794 (35.46%)	145 (67.44%)	
No	10,547 (64.54%)	70 (32.56%)	
**Alcohol status, N**			0.063
Yes	8,917 (54.57%)	131 (60.93%)	
No	7,424 (45.43%)	84 (39.07%)	
**BMI (kg/m^2)**	28.10 (24.04, 33.30)	29.30 (23.98, 33.97)	0.293
**Hypertension, N**			0.020
Yes	5,419 (33.16%)	88 (40.93%)	
No	10,922 (66.84%)	127 (59.07%)	
**diabetes mellitus, N**			0.999
Yes	1,759 (10.76%)	23 (10.70%)	
No	14,582 (89.24%)	192 (89.30%)	

Supplemental Materials

Table S3 Baseline characteristics of participants with and without ovary cancer.

**Variables**	**Non-ovary cancer** (N = 16,484)	**Ovary cancer** (N = 72)	***P*-value**
**OBS**	17.00 (12.00, 23.00)	14.50 (10.00, 21.00)	0.005
**Age (years)**	46.00 (33.00, 62.00)	62.00 (46.50, 72.00)	<0.001
**Race/ethnicity, N**			0.239
Mexican American	2,894 (17.56%)	14 (19.44%)	
Non-Hispanic Black	3,404 (20.65%)	13 (18.06%)	
Non-Hispanic White	7,462 (45.27%)	33 (45.83%)	
Other Hispanic	1,382 (8.38%)	10 (13.89%)	
Other Race	1,342 (8.14%)	2 (2.78%)	
**Family poverty ratio, N**			0.911
Low	5,075 (30.79%)	23 (31.94%)	
Median	6,211 (37.68%)	28 (38.89%)	
High	5,198 (31.53%)	21 (29.17%)	
**Education level, N**			0.042
Below high school	3,836 (23.27%)	25 (34.72%)	
High school	3,636 (22.06%)	17 (23.61%)	
Above high school	9,012 (54.67%)	30 (41.67%)	
**Marital status, N**			0.089
Married	8,711 (52.85%)	45 (62.50%)	
Living with partner	1,165 (7.07%)	1 (1.39%)	
Living alone	6,608 (40.09%)	26 (36.11%)	
**Smoking status, N**			0.461
Yes	5,910 (35.85%)	29 (40.28%)	
No	10,574 (64.15%)	43 (59.72%)	
**Alcohol status, N**			0.477
Yes	9,012 (54.67%)	36 (50.00%)	
No	7,472 (45.33%)	36 (50.00%)	
**BMI (kg/m^2)**	28.10 (24.03, 33.30)	29.52 (25.73, 33.84)	0.158
**Hypertension, N**			<0.001
Yes	5,467 (33.17%)	40 (55.56%)	
No	11,017 (66.83%)	32 (44.44%)	
**diabetes mellitus, N**			0.011
Yes	1,767 (10.72%)	15 (20.83%)	
No	14,717 (89.28%)	57 (79.17%)	

Supplemental Materials

Table S4 Baseline characteristics of participants with and without uterine cancer.

Variables	Non-uterine cancer (N = 16,423)	Uterine cancer (N = 133)	*P*-value
**OBS**	17.00 (12.00, 23.00)	16.00 (12.00, 22.00)	0.211
**Age (years)**	46.00 (33.00, 62.00)	62.00 (51.00, 71.00)	<0.001
**Race/ethnicity, N**			0.101
Mexican American	2,885 (17.57%)	23 (17.29%)	
Non-Hispanic Black	3,401 (20.71%)	16 (12.03%)	
Non-Hispanic White	7,423 (45.20%)	72 (54.14%)	
Other Hispanic	1,379 (8.40%)	13 (9.77%)	
Other Race	1,335 (8.13%)	9 (6.77%)	
**Family poverty ratio, N**			0.099
Low	5,049 (30.74%)	49 (36.84%)	
Median	6,186 (37.67%)	53 (39.85%)	
High	5,188 (31.59%)	31 (23.31%)	
**Education level, N**			0.019
Below high school	3,818 (23.25%)	43 (32.33%)	
High school	3,621 (22.05%)	32 (24.06%)	
Above high school	8,984 (54.70%)	58 (43.61%)	
**Marital status, N**			0.840
Married	8,683 (52.87%)	73 (54.89%)	
Living with partner	1,156 (7.04%)	10 (7.52%)	
Living alone	6,584 (40.09%)	50 (37.59%)	
**Smoking status, N**			0.018
Yes	5,878 (35.79%)	61 (45.86%)	
No	10,545 (64.21%)	72 (54.14%)	
**Alcohol status, N**			<0.001
Yes	8,997 (54.78%)	51 (38.35%)	
No	7,426 (45.22%)	82 (61.65%)	
**BMI (kg/m[2])**	28.10 (24.03, 33.30)	28.85 (25.23, 35.40)	0.105
**Hypertension, N**			<0.001
Yes	5,438 (33.11%)	69 (51.88%)	
No	10,985 (66.89%)	64 (48.12%)	
**diabetes mellitus, N**			0.005
Yes	1,757 (10.70%)	25 (18.80%)	
No	14,666 (89.30%)	108 (81.20%)	
